# Ion bombardment induced buried lateral growth: the key mechanism for the synthesis of single crystal diamond wafers

**DOI:** 10.1038/srep44462

**Published:** 2017-03-15

**Authors:** Matthias Schreck, Stefan Gsell, Rosaria Brescia, Martin Fischer

**Affiliations:** 1 Universität Augsburg, Institut für Physik, D-86135 Augsburg, Germany

## Abstract

A detailed mechanism for heteroepitaxial diamond nucleation under ion bombardment in a microwave plasma enhanced chemical vapour deposition setup on the single crystal surface of iridium is presented. The novel mechanism of **Io**n **B**ombardment **I**nduced **B**uried **L**ateral **G**rowth (IBI-BLG) is based on the ion bombardment induced formation and lateral spread of epitaxial diamond within a ~1 nm thick carbon layer. Starting from one single primary nucleation event the buried epitaxial island can expand laterally over distances of several microns. During this epitaxial lateral growth typically thousands of isolated secondary nuclei are generated continuously. The unique process is so far only observed on iridium surfaces. It is shown that a diamond single crystal with a diameter of ~90 mm and a weight of 155 carat can be grown from such a carbon film which initially consisted of 2 · 10^13^ individual grains.

Synthesis of monocrystalline diamond in wafer size is a fascinating challenge due to a wide range of interesting applications that require high quality crystals to achieve the ultimate device performance as predicted from the intrinsic material parameters. Crystals from natural sources can neither fulfil the requirements with respect to reproducible quality nor to the size of available samples. The latter limitation still holds also for crystals synthesized by the classical high pressure high temperature (HPHT) method.

The development of chemical vapour deposition (CVD) methods has removed the severe size restrictions. In the meantime, polycrystalline layers with an area of 0.5 m^2^ mark the state of the art for the hot filament (HF) technique^1^. Towards wafer-size single crystals, two different concepts are intensively explored. A first approach utilizes homoepitaxial deposition on HPHT substrates. Meanwhile, 2-inch size samples have been obtained by combination with cloning and overgrowth of tiled substrates as reported by Yamada *et al*.^2^. However, these still contain internal grain boundaries.

A second concept is based on heteroepitaxy – a common technique that is commercially applied on large scale for various materials of high technological relevance like GaN^3^. For diamond heteroepitaxy, two principal demands have to be fulfilled, namely finding the optimum substrate and the appropriate nucleation method. Both have been clearly identified: bias enhanced nucleation (BEN) using a negative DC voltage^4^ is the most powerful nucleation technique and iridium is the unrivalled substrate.

A huge number of studies have been performed trying to figure out the physical reasons for iridium’s uniqueness^5^,^6^,^7^,^8^. Basically, a variety of alternative substrate materials also facilitate generation of oriented diamond grains (Si, SiC, Cu, Ni, Re, TiC). Compared to Ir, on all the alternative substrates the density of epitaxial diamond grains was significantly lower and their orientation distribution was much broader. As a consequence, even extended growth never turned the epitaxial films into real single crystals (for reviews see refs 9 and 10).

In the field of heteroepitaxial growth of compound semiconductors, fit in lattice parameter is typically an important issue for the optimization of structural quality (see a review on substrates for GaN heteroepitaxy in ref. 3). According to this consideration, the fcc metals Ni or Cu should be much better growth substrates for diamond than the fcc metal Ir ((a_Dia_-a_Metal_)/a_Metal_ for Ni: +1.2%, Cu: −1.3% Ir: −7.1%). Since all experimental results reported up to now completely contradict this simple expectation, other factors are apparently of higher relevance.

The particularities of BEN on iridium like the pattern formation (“domains”) provide the key to unravel the mystery. The phenomena were consistently described by many groups but a convincing explanation has not been found up to now. In the present work we will demonstrate that invariably all the experimental observations can be consolidated in one comprehensive model. This model comprises a novel process in which the vast majority of isolated epitaxial diamond grains are not formed in independent real nucleation events but by buried lateral growth within a ~1 nm thick carbon matrix induced by intense ion bombardment. We will also show that this concept can provide the basis for the synthesis of single crystal diamond with unrivalled dimensions and a weight of more than 100 carat.

## Results

**Figure 1:**
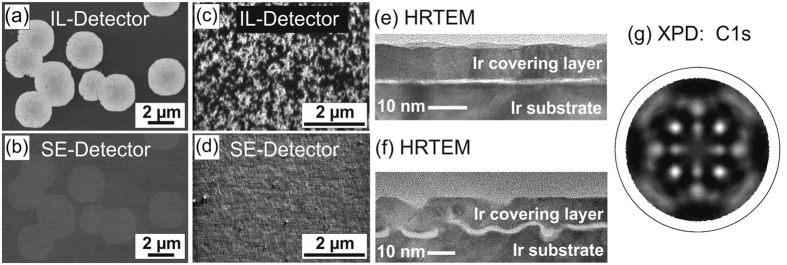
Major characteristics of diamond nucleation layers formed by BEN on Ir(001) surfaces. (**a**) SEM image of the Ir surface after 10 min BEN in a pure DC discharge (100 mbar, 2% CH_4_/H_2_, U_Bias_: about −280 V) taken with IL detector. (**b**) Identical spot imaged with SE detector. (**c**) Highly branched fractal like domains on Ir surface after BEN (30 mbar, 7% CH_4_/H_2_, U_Bias_: about −280 V) imaged with IL detector (**d**) identical spot imaged with SE detector. (**e**) Cross section HRTEM image of a BEN layer sandwiched between the non-roughened Ir substrate and a ~10 nm thick Ir covering layer. (**f**) Cross section HRTEM image of a BEN layer on a roughened Ir substrate. (**g**) XPD pattern of the C1s electrons emitted from a BEN layer. (XPD pattern: Reprinted from *Diamond Relat. Mater.* **17**^12^.

Figure 1 summarizes the dominant phenomenological features of the nucleation layer formed by BEN on iridium as revealed in numerous former studies^11^,^12^,^13^. The first is the appearance of bright areas in scanning electron microscopy (SEM) images taken by the in-lens (IL) detector which is specifically sensitive to work function contrast. These areas usually called “domains” are unequivocally correlated to the diamond nucleation centres as substantiated by subsequent growth experiments on the same spot of the sample^7^. Depending on the specific BEN conditions, the patterns vary from compact homogeneous areas with smooth edges (Fig. 1(a)) to highly branched fractal-like structures (Fig. 1(c)). The contrast in the corresponding images taken with the standard secondary electron detector (SE) specifically sensitive to topographic contrast indicates negligible height effects (see Fig. 1(b) and (d)). Figure 1(e) and (f) are cross section high resolution transmission electron microscopy (HRTEM) images of two samples after nucleation. The bright slit substantiates the existence of a continuous ~1 nm thick carbon layer on a flat (Fig. 1(e)) or roughened (Fig. 1(f)) iridium substrate. In both cases the Ir crystal lattice is clearly resolved in the substrate and the covering layer deposited before TEM sample preparation as a protection and for a better visualization of the BEN layer. Inside the carbon film, crystalline structures ascribable to diamond have not been detected by this technique.

Further studies on the nature of the carbon layer inside the domains revealed that it is thinner and harder, containing a higher sp^3^ fraction than outside^14^. First direct evidence for the existence of diamond structures within the domains were then obtained from spectral features in spatially resolved X-ray absorption near edge spectroscopy (XANES) measurements^15^.

The most significant information, however, was derived from X-ray photoelectron diffraction (XPD) patterns (see Fig. 1(g)). In sharp contrast to the TEM results, these patterns clearly prove the presence of crystalline diamond in the BEN layer^12^. Their quantitative evaluation facilitates also an estimation of the fraction of carbon atoms located in an ordered environment. With the simple assumption that all carbon atoms contributing to the pattern are located in nuclei, i.e. isolated diamond crystals embedded in an amorphous carbon matrix, a value of ~13 nm is calculated for their lateral size^12^. Diamond crystals of this size could easily be detected by HRTEM. However, lattice images compatible with diamond have never been found by us in spite of many HRTEM studies on a large number of BEN samples.

**Figure 2:**
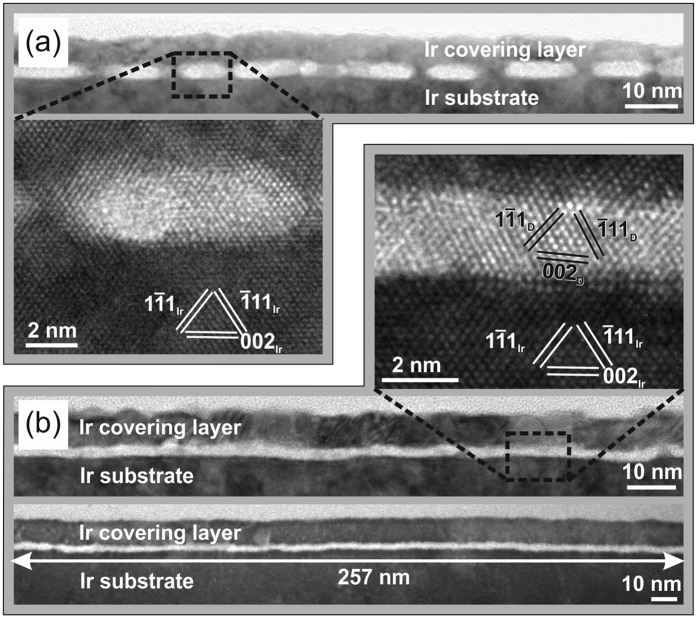
Cross section HRTEM micrographs of diamond grains formed during 5 s growth after termination of the BEN treatment (40–45 min). The ~10 nm covering layer was deposited before TEM sample preparation. Both samples were deposited under virtually identical conditions T_Substrat_ = 730–750 °C, 7% CH_4_/H_2_, U_Bias_ = −260 V. In (**a**) isolated grains had formed while in (**b**) a 257 nm wide single crystal layer is observed.

As a consequence, a conclusive nucleation model has to resolve this fundamental contradiction as well as explain the unique pattern formation that forces the diamond nuclei on Ir to agglomerate in well-defined domain areas. In the following we will focus on some particularly interesting TEM observations on the carbon layer that formed immediately after switching off the bias voltage, i.e. growth occurs via standard CVD without ion bombardment. It turned out that already 5 s after BEN essentially all amorphous carbon is removed and crystalline diamond structures have developed. The cross section TEM image in Fig. 2(a) shows isolated crystallites with a typical distance of 15–20 nm, a height of 2 nm and no amorphous carbon in between. This observation was confirmed in several further samples grown for 5, 10, 20 and 60 s after BEN which showed how initially isolated grains progressively coalesced with increasing time^16^,^17^.

**Figure 3:**
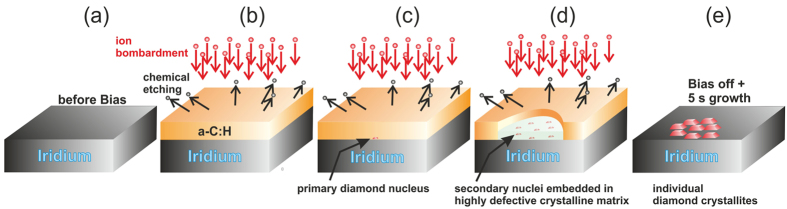
Schema describing the typical phenomenology of BEN on Ir. (**a**) Ir surface exposed to plasma before biasing. (**b**) The a-C:H layer formed after start of the BEN process. Dynamic equilibrium between deposition of hyperthermal particles and etching by atomic hydrogen limits layer thickness to a constant value of 1–2 nm. (**c**) Spontaneous formation of a primary diamond nucleus. (**d**) Lateral expansion of the domain and formation of secondary nuclei embedded in a highly defective crystalline matrix (domain) which is thinner than the surrounding a-C:H film. (**e**) During the first 5 s after termination of BEN the atomic hydrogen etches completely the a-C:H precursor phase as well as the defective matrix in the domains. Simultaneously crystalline diamond grains with a height of 2 nm at a distance of 15–20 nm are formed.

All the experimental results described up to now can be summarized in the phenomenological schema plotted in Fig. 3. After start of the BEN treatment the initially bare Ir surface (a) is quickly covered by an amorphous hydrogenated carbon (a-C:H) layer (b). Its thickness of 1–2 nm is largely independent of biasing time and results from a dynamic equilibrium between deposition of hyperthermal species and chemical etching by atomic hydrogen. Then spontaneous nucleation occurs (c). It is quite a rare event: the regular shape of the domains in Fig. 1(a) facilitates the estimation that only 8–10 primary nuclei have been generated within 10 min in an area of 140 μm^2^ (equivalent to a nucleation rate of ~1 · 10^−4^ s^−1^ μm^−2^). In their close proximity, generation of secondary nuclei immediately starts leading to a continuous growth of the domains (d). Five seconds after the end of the BEN process, the amorphous carbon is completely etched and isolated tiny crystals with a height of 2 nm cover the Ir surface (e). With a typical crystallite density of 3 · 10^11^ cm^−2^ as derived by TEM and AFM we calculate a ratio of 2 · 10^4^ between secondary and primary nucleation events for the domains in Fig. 1(a).

The descriptive schema in Fig. 3 can neither explain the generation of the primary nor of the secondary nuclei. In heterogeneous catalysis, spatial pattern formation can result from self-organization based on autocatalytic processes^18^. Transferring this concept to agglomeration of the diamond grains on Ir, i.e. to the generation of secondary nuclei, would require a reduction of the nucleation barrier in the neighbourhood of existing nuclei. However, finite element simulations recently revealed that the stress field emanating from pseudomorphic diamond crystals via the Ir substrate would rather suppress further nucleation than cause a positive feedback^19^.

The key indication was finally obtained from the new sample in Fig. 2(b). The cross section TEM image shows a continuous single crystal diamond layer with a height of 1.8 nm and a width of 257 nm which had formed during the first 5 s after termination of BEN on a flat Ir surface. Due to the short growth time, formation of the continuous layer by coalescence of individual islands can be ruled out. Instead, the present observation yields compelling arguments for the existence of a crystalline connection inside the carbon layer over the whole lateral distance at the end of the ion bombardment stage. We interpret this finding as the decisive proof for the involvement of lateral diamond growth as the crucial process in the domain formation.

**Figure 4:**
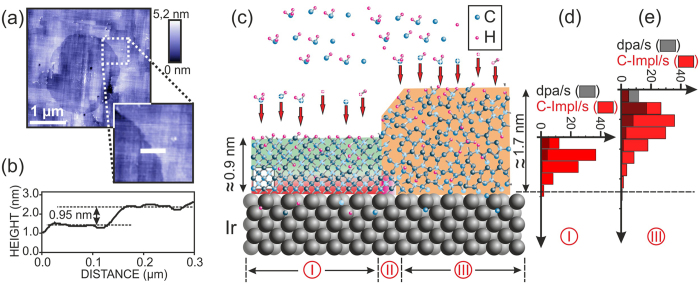
Detailed description of the IBI-BLG mechanism. (**a**) AFM image and (**b**) height profile of the sample shown in Fig. 1(a). (**c**) Schema of the layer structure corresponding to the edge of the domain shown in (**a**). The white square indicates the size of a diamond unit cell. The domain region “I” is crystalline with high defect density which strongly increases from the Ir interface to the free surface (colour code identical to Fig. 3). Only the first carbon layers (red) will survive the H etching after BEN and act as nucleus. The region “III” displays the amorphous a-C:H precursor film. Region “II” is the reaction zone where ion bombardment driven lateral growth occurs. During growth of the domain, region “II” moves to the right. (**d**,**e**) Simulated depth profiles for carbon implantation normalized to the atomic density and displacements of target carbon atoms per carbon target atom per second (dpa/s) in regions “I” and “II”, respectively. The grey bars of the displacements are semi-transparent in the foreground.

For the further discussion of a mechanism we consider the simplest situation of homogeneous domains with smooth edges on non-roughened iridium as shown in Fig. 1(a). Atomic force microscopy (AFM) data of a single domain on this sample are shown in Fig. 4(a). Crossing the domain edge from inside to outside (see line scan in Fig. 4(b)), an upwards step is measured with an average height of 0.83 ± 0.14 nm as deduced from 13 individual line scans. In cross section TEM images of this sample, different regions with ~0.9 nm and ~1.6 nm thickness have been found. We interpreted them in terms of domain and a-C:H areas, respectively.

All the described observations can be condensed into the model presented in Fig. 4(c). The domain region “I” on the left-hand side consists of a highly defective diamond crystal with a thickness of ~2.5 unit cells (0.9 nm). Its defect density - high enough to prevent any lattice imaging in HRTEM studies - increases from the Ir interface to the free surface. At the same time the defect density is low enough to facilitate clear C1s XPD patterns (Fig. 1(g)). This ostensible contradiction can be easily resolved by the facts that atomic resolution in HRTEM requires an ordered structure over a couple of lattice planes while XPD pattern formation is dominated by forward scattering of photoelectrons by the next nearest neighbours of the emitting atoms^20^. The latter process requires only a short range order.

In region “I” only the lower few atomic layers directly above the Ir substrate are structurally perfect and stable enough so that they can act as nucleus. In contrast, the upper part of the layer is identical to the “highly defective crystalline matrix” in Fig. 3(d). This matrix is unstable towards etching by atomic hydrogen after termination of BEN.

Region “III” on the right-hand side consists of an amorphous a-C:H layer without any crystalline diamond structures. This region generates featureless C1s XPD patterns^12^. From the size of the domain in Fig. 4(a) an increase in diameter of 12 μm/h is deduced (assuming that the domain was nucleated in the first minutes of the BEN treatment). Thus, the reaction zone “II” highlighted in red moved with 6 μm/h (1.67 nm/s) to the right.

In a first approach to explain the lateral expansion of the domains one may consider standard CVD growth. This occurs directly at the film/gas interface and involves the creation of dangling bonds by abstraction of hydrogen from the hydrogen stabilized surface followed by adsorption of hydrocarbon radicals from the gas phase. The carbon atom of this hydrocarbon species is then incorporated step by step into the diamond crystal lattice.

In former bias assisted growth studies it was actually observed that a soft negative bias can be compatible with crystalline growth. However, the increasing ion bombardment caused permanent crystal damage which manifested itself in a rapid degradation of the mosaic spread^21^. The present ion bombardment occurring during BEN on Ir is significantly harsher – it suppresses vertical crystal growth completely^22^. As a consequence, lateral surface growth under ion bombardment would never yield the excellent epitaxial alignment of diamond films that is invariably reported by all groups for heteroepitaxy on Ir^23^,^24^,^25^.

On the other hand, below the surface and in particular directly at the iridium interface, growth occurs without direct contact to the gas phase. Thus, surface growth mechanisms can be ruled out completely and lateral expansion is based on pure solid state processes controlled by implanted hyperthermal species. In order to highlight the specific character of this new process we suggest the term **I**on **B**ombardment **I**nduced - **B**uried **L**ateral **G**rowth (IBI-BLG).

For a further substantiation of this model the atomistic effects of the bombardment by hyperthermal particles have to be considered. They are twofold: first, the projectiles can displace target atoms and second, they are stopped in the solid. Lifshitz, Lee, Frauenheim *et al*. have studied diamond nucleation from energetic species^26^,^27^,^28^. They used BEN on Si for their experiments and performed molecular dynamic simulations. They proposed that spontaneous precipitation of pure sp^3^ clusters occurs in the dense a-C:H matrix with few of them (1 in 10^4^ to 10^6^) being perfect diamond clusters^26^. The interface with a foreign crystalline material can stabilize clusters with heteroepitaxial alignment and increase the probability for their formation due to a “mold” effect^27^. They suggested that the increase in size from ~30 to ~10^4^ atoms per cluster is then driven by ion bombardment induced preferential displacement processes^26^ as described by Banhart *et al*.^29^,^30^,^31^,^32^.

The ideas presented in refs 26 and 27 provide a plausible model that can explain the generation of primary nuclei in our experiments on Ir. However, the domain formation over micron size lateral distances within few minutes without twinning or secondary nucleation requires further in-depth considerations. We therefore modelled the concrete layer structure of Fig. 4 by Monte Carlo (MC) simulations using SRIM^33^ in order to derive displacement and implantation profiles. As an input we needed (a) the layer structure (specifically the density and displacement energies) and (b) the particle current densities.

For the density of the domain region “I” we assumed 90% of the value of a perfect diamond crystal (i.e. 1.6 · 10^23^ C-at/cm^3^). From the AFM step height (0.83 nm) and the ratio in carbon coverage deduced by Auger electron spectroscopy AES (20% higher coverage in region “III” than in “I”)^14^ we calculated a density of 1.0 · 10^23^ C-at/cm^3^ (i.e. 2.0 g/cm^3^) for region “III”. This value is typical for a-C:H layers^34^.

During the BEN treatment on Ir we measure a typical discharge current density of 50 mA/cm^2^. Right above the cathode the discharge current is primarily carried by ions. Thus, an ion particle flux density of 3 · 10^17^ s^−1^cm^−2^ hits the film surface. Kátai *et al*. have measured energy and flux of H^+^, H_2_^+^, H_3_^+^, C^+^, CH^+^, CH_2_^+^, CH_3_^+^, CH_4_^+^, C_2_^+^, C_2_H^+^, C_2_H_2_^+^, C_2_H_3_^+^ by mass spectroscopy during BEN on Si^35^. To deduce approximate values for the situation on Ir, we multiplied the energy data by 1.4 due to the higher bias voltage (−280 V instead of −200 V) and scaled all particle fluxes by a constant factor to reconcile the total ion flux with the measured biasing current. This procedure provided the input data for an estimation of carbon implantation and displacement profiles by SRIM. For the displacement threshold of carbon atoms in the domain area “I” and the precursor area “III” we used the values 35 eV and 20 eV respectively. Figure 4(d) and (e) show the obtained depth profiles of displacements of target carbon atoms per target carbon atom per second (dpa/s) and of implanted carbon atoms per second (C-Impl/s). The profiles of implanted carbon atoms are normalized to the atom density in the respective region. A value of 1 C-Impl/s means that all carbon atoms at this depth are exchanged once per second. For both regions the implantation peak corresponds to a maximum exchange rate of ~40 s^−1^. The height of the displacement profiles is generally lower - except for the first value in (e) - and they are shifted to lower depth.

In the work of Banhart *et al*. with transmitting high energy particles only displacements played a role. They could show that this bombardment cannot only be destructive for a crystalline solid but in contrary, graphitic or even amorphous carbon can be transformed to crystalline diamond under appropriate flux, energy and especially temperature conditions. They attributed the effect to the lower displacement threshold for carbon atoms in graphite as compared to diamond and derived a non-equilibrium phase diagram of the diamond-graphite system under particle irradiation^32^. According to this diagram, the stability region of diamond at the temperature of our BEN processes (~1000 K) requires an irradiation intensity Φ above 10^−4^ dpa/s in graphite. Comparison with Fig. 4(e) reveals that this condition is fulfilled over the whole thickness range. Even at the Ir interface the obtained value of ~0.1 dpa/s is three orders of magnitude above the threshold.

As a further plausibility check, the propagation velocity of the domain boundary is compared with Banhart’s work. From their measurements and predictions in ref. 32, a lateral velocity of the diamond/graphite phase boundary equivalent to 0.05 nm/displacement can be extracted. To explain our lateral domain growth velocity of 1.67 nm/s (see Figs 1(a) and 4(a)), one would need an irradiation intensity that generates ~36 dpa/s. However, even in the damage peak our MC simulations predict only 17 dpa/s. Towards the Ir interface the values decrease to 0.1 dpa/s.

As a second effect, the depth profiles of implanted carbon projectiles (red bars in Fig. 4(d,e)) are considered. Both profiles are normalized to the atom density in the corresponding region. They show that the carbon atoms in the implantation peaks are replaced with a frequency of ~40 s^−1^. Thus, implantation apparently plays the dominant role for the phase transformation. The integral carbon deposition rate (which is compensated by an identical etching rate in the dynamic equilibrium) is equivalent to an effective exchange of the whole film every ~0.07 s.

Close to the Ir interface in region “III” the exchange rate (including the C-atoms that diffuse back from the Ir) is ~2.5 C-Impl/s. With this value a lateral growth rate per implanted carbon atom of 1.67 nm/s/2.5 C-Impl/s = 0.67 nm/C-Impl is calculated. At the transition to the domain area (i.e. the reaction zone “II”) the gradual drop in carbon coverage as derived from AES yields a further factor of 2 (in C-Impl/s) so that we end up with a value of 0.3–0.4 nm/C-Impl.

From these results, we first conclude that carbon implantation events (0.3–0.4 nm/C-Impl) are apparently by a factor of 6–8 more efficient in promoting phase transformation than the displacements considered in ref. 32 (0.05 nm/displacement). Their lower efficiency may be attributed to the statistical fact that a high fraction of carbon atoms is kicked away from the diamond/a-C:H interface. Second, taking into account the atomic volume of carbon in diamond (0.178 nm)^3^, the simulation result can be summarized in the simple statement that for every carbon atom that arrives at the interface by ballistic transport the domain grows laterally by two carbon cells (2 · 0.178 nm). The implanted carbon atom first increases the local atom density. Then the elevated process temperature in combination with the excess kinetic energy of the projectile provide the activation that allows the implanted C atom and potentially one or few nearest neighbour atoms to relax into the diamond structure and cause the lateral growth of the diamond lattice.

Our simulations yield plausible numbers for a subsurface crystal growth driven by implanted carbon atoms which is controlled and stabilized by the iridium surface. The IBI-BLG mechanism provides a straightforward explanation for the common observation that all diamond grains generated by BEN generally feature an excellent epitaxial alignment. Furthermore, we attribute iridium’s uniqueness for this process on the one hand to the strong Ir-C binding at the interface which manifests itself also in the excellent adhesion of the diamond layers even after several days in the CVD reactor. On the other hand, we suppose that its missing affinity to carbide formation and the negligible bulk solubility of carbon^36^ are further crucial ingredients.

Finally, we discuss the reasons for the extremely rare appearance of an extended 2D nucleation layer in contrast to the usually observed isolated nucleation centres. As shown in Fig. 1, the shape of the domains and the modification of the Ir surface are very sensitive to the local bombardment conditions. In addition, domains can grow or shrink in regions few millimetres apart on one sample^11^. Eres *et al*.^37^ reported a narrow bias voltage window: nucleation was completely absent below or above a certain voltage. All these experimental findings are manifestations of the same critical property: diamond nucleation and domain formation require rather well defined BEN conditions. Within this parameter window the 2D layer represents a metastable state with an even narrower range of bombardment conditions. One possible driving force for the splitting into isolated secondary nuclei during IBI-BLG could be the relaxation of coherence strain. For thin pseudomorphically grown diamond layers on Ir this effect has formerly been studied by finite element (FE) simulation studies^19^.

In the supplementary information further MC type simulations are described which reproduce the different shapes of the domains by variation of few simple parameters.

**Figure 5:**
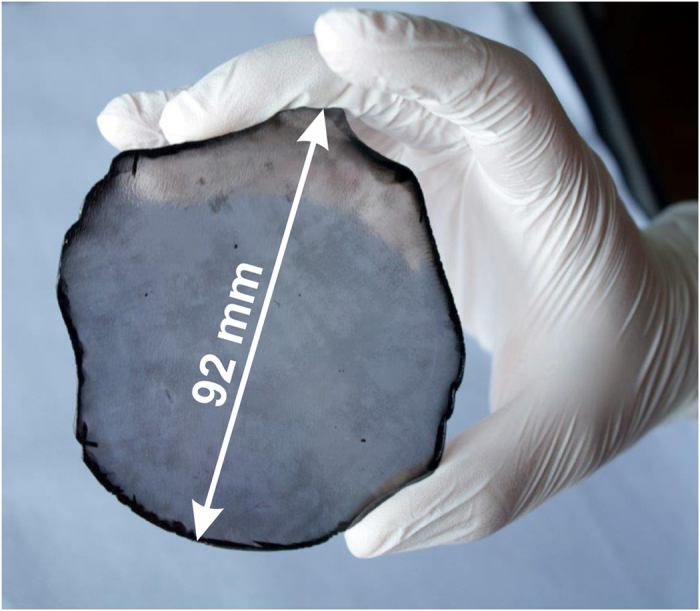
Freestanding unpolished diamond single crystal synthesized by heteroepitaxy on Ir/YSZ/Si(001). The thickness of the disc is 1.6 ± 0.25 mm and its weight is 155 carat. Overall 43 X-ray diffraction (XRD) rocking curves for the Dia(004) reflection were measured along two perpendicular directions across the wafer (see supplementary information). The average full width at half maximum (FWHM) was 0.064 ± 0.011°, a value typical for IIa crystals^41^. For the corresponding azimuthal scans (Dia(311)) we obtained 0.12 ± 0.04°. In addition, μ-Raman measurements at 11 arbitrary spots across the wafer yielded an average FWHM of 1.75 ± 0.07 cm^−1^.

In spite of the apparently narrow process window for BEN on Ir, the technique can be mastered even for large area substrates. Figure 5 shows a freestanding 155 carat diamond plate with a thickness of 1.6 ± 0.25 mm grown heteroepitaxially on Ir/YSZ/Si(001). It was formed by the coalescence of ~2 · 10^13^ individual grains which had evolved from secondary (and few primary) nuclei after BEN on the 4-inch iridium surface. The dislocation density of heteroepitaxial diamond on Ir after 1.5 mm growth is typically ~4 · 10^7^ cm^−2^, i.e. significantly smaller than for standard IIa crystals (10^8^–10^9^ cm^−2^)^38^. More details on the structural quality and homogeneity are given in the figure caption and the supplementary.

In summary, during the two decades after the pioneering work^5^ by Sawabe and colleagues in Japan (1996), a large variety of puzzling observations related to BEN on Ir has been reported by different groups. The present work is an attempt to assemble all pieces of the puzzle into a consistent model based on new HRTEM observations and MC simulations. This model provides an impressing proof that ion bombardment cannot only be used to tailor nanostructured carbon materials^39^. Instead, it can apparently also serve as a basis for the synthesis of wafer-size single crystal diamond on Ir. The successful demonstration of heteroepitaxial diamond wafers is supposed to remove one crucial technological hurdle that existed so far for the realization of electronic devices and other high-end diamond applications.

## Methods

Multilayers of the structure Ir/SrTiO_3_(001) and Ir/YSZ/Si(001) (with YSZ = yttria stabilized zirconia) were used as substrates for the BEN experiments^22^,^40^. BEN and growth were performed in a microwave plasma CVD setup at temperatures of 700–800 °C, with 5–10% CH_4_ in H_2_ at a pressure of 30 mbar and for a microwave power of 1100 W. Additionally, some nucleation experiments were performed in a pure DC discharge setup at 100 mbar and 2% CH_4_. The BEN voltage applied to the substrate was in the range of −250 to −300 V.

The wafer shown in Fig. 5 was nucleated in a specifically designed setup which is capable of both, growth and nucleation, combining microwave excitation and DC discharge. The bias current density was about 50 mA/cm^2^. During growth under high power conditions (915 MHz) 8% CH_4_ in H_2_ and few ppm of nitrogen were used in the gas phase for a time of about 5 days.

All samples were first examined with a LEO DSM 982 Gemini SEM equipped with an in-lens (IL) secondary electron detector, which is sensitive to work function differences. The topography of the samples was examined by an AutoProbe CP Research atomic force microscope (AFM) operated in non-contact mode. TEM investigations were performed using a JEOL field-emission microscope JEM-2100F operated at 200 kV.

## Additional Information

**How to cite this article:** Schreck, M. *et al*. Ion bombardment induced buried lateral growth: the key mechanism for the synthesis of single crystal diamond wafers. *Sci. Rep.* **7**, 44462; doi: 10.1038/srep44462 (2017).

**Publisher's note:** Springer Nature remains neutral with regard to jurisdictional claims in published maps and institutional affiliations.

## Supplementary Material

Supplementary Information
