# Supplementary material for: Ion bombardment induced buried lateral growth: the key mechanism for the synthesis of single crystal diamond wafers
Source: Sci Rep. 2017 Mar 15;7:44462. doi: 10.1038/srep44462 (PMC5353677; doi:10.1038/srep44462)
Supplement: Supplementary Information [file srep44462-s1.pdf]

SUPPLEMENTARY INFORMATION

Ion bombardment induced buried lateral growth: the key mechanism for the synthesis of single crystal diamond wafers

M. Schreck*, S. Gsell, R. Brescia[1], M. Fischer

Universität Augsburg, Institut für Physik, D-86135 Augsburg, Germany
**Corresponding author: matthias.schreck@physik.uni-augsburg.de*

[1]*Current address: Department of Nanochemistry, Istituto Italiano di Tecnologia, I-16163 Genoa, Italy*

1. Monte Carlo simulations of the domain shapes

A crucial additional check for the validity of our model consists in its ability to describe the formation of the different shapes of the domains as we find them in our experiments. They comprise compact circular or nearly square-shaped patterns with smooth edges, corral like structures with the central part nearly free of epitaxial diamond up to highly branched fractal shaped structures (see Fig. 1). Figure 1 also shows crystal islands that evolve in a short CVD growth step from the different types of domains.

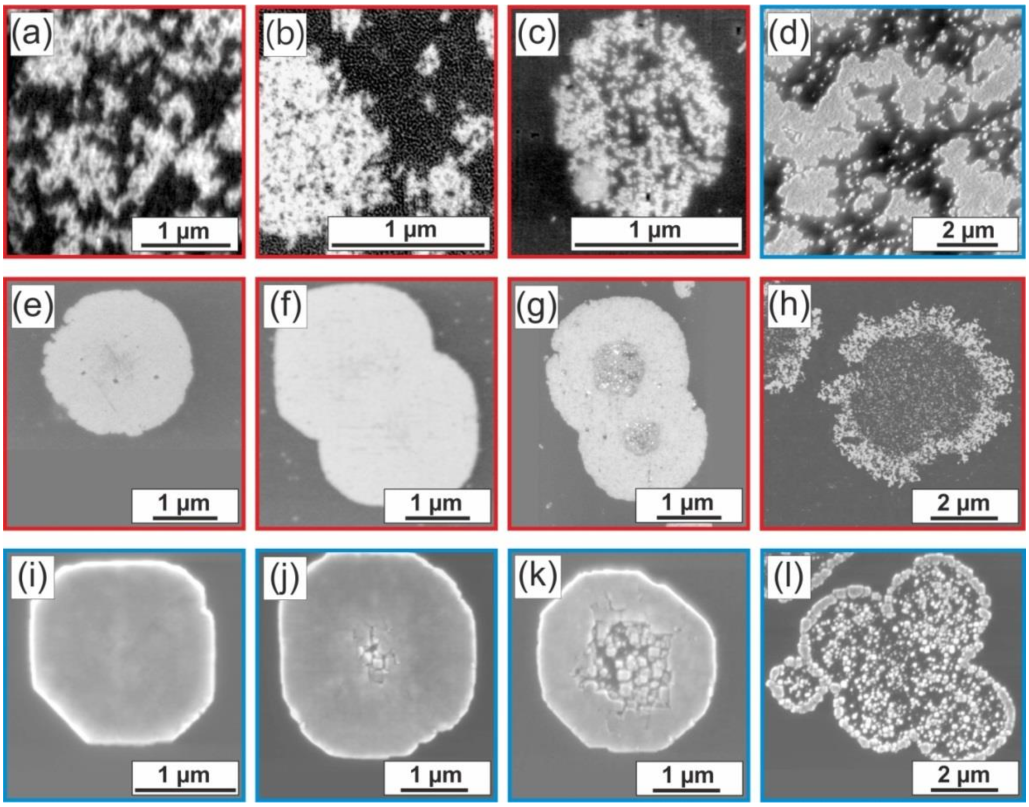

Figure 1: Variation of domain shape as observed after BEN (red frame) and after BEN plus a 15-min growth step (blue frame). (a) – (d) Highly branched fractal like domains. (e) – (l) Smooth circular and rectangular domains with gradual transition to corral structure. Micrographs (i) – (l) are taken from different regions of the identical sample.

Similar variations in shape of islands ranging from regular polygons to dendritic fractal patterns have been observed in experiments with a variety of thin layer systems like sub-monolayer growth of metals[1,2], or the synthesis and etching of intrinsically 2D material systems, e.g. graphene[3] or h-BN[4]. The highly branched shape is typically interpreted as a non-equilibrium state which is caused by kinetic constraints (see for example the diffusion-limited-aggregation (DLA) growth mechanism[1]).

For a better understanding of the mechanisms which generate the large variety of self-organized domain patterns during BEN on Ir, a second type of MC simulations was performed. It is based on two fundamental observations:

1) Domains can increase in size and shrink on different spots of the same sample only due to slight differences in the local ion bombardment conditions[5]. Thus, there is a kinetic competition between lateral spreading of the crystalline diamond regions along the Ir interface (transformation amorphous-to-crystalline) and the local destruction of crystallinity (back-transformation crystalline-to-amorphous).
2) A continuous 2D layer is a rare metastable situation. Normally, isolated grains with a distance of 15 - 20 nm are found after BEN and a short growth step (see Fig. 2(a) and schema in Fig. 3(e) in the main article). This distance corresponds to a nucleation density of $3\cdot10^{11}$cm^{-3} in good agreement with reports from other groups[6,7,8].

We start with a two-dimensional matrix (201x201) of square cells (Fig. 2). The grid spacing of the matrix corresponds to a distance of 18 nm between nuclei in the real experiment. Thus, the simulated region is equivalent to an area of 3.6 x 3.6 µm^2 similar to the regions shown in Fig. 1. Each cell can have two possible states N = 0 (no nucleus) and N =1 (nucleus). The transition "0→1" is a nucleation event, the transition "1→0" is a destruction event.

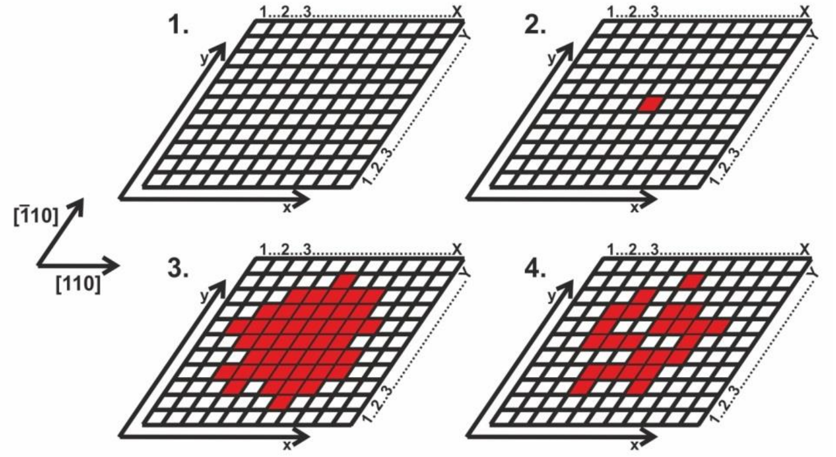

Figure 2: Schema of consecutive steps in the MC simulation: 1) creation of the matrix 2) placement of the first nucleus in the centre of the matrix 3) + 4) lateral spread.

In the subsequent simulation the cells of the matrix are selected in a stochastic process. In case the state of the chosen cell is "0", it can be changed to "1" with a certain nucleation probability p_N using a random number generator. Switching from "0" to "1" of an initially empty cell can only occur when one of the neighbouring cells is in state "1", i.e. lateral spread only works via direct neighbours. The probability for a "0→1" transition of a cell is given by the contributions of all neighbouring cells with state "1" (see Eq. (1)). The control parameter

“S” is called the nucleation strength. The physical background for the last assumption in the real IBI-BLG process is that carbon atoms in the amorphous region are the easier transformed to crystalline diamond the more direct neighbours are already located in the ordered lattice structure. Diagonal and off-diagonal neighbours are distinguished to account for crystal anisotropy with the parameter “A”.

When an occupied cell in the state “1” is selected in the stochastic process it can be destroyed (“1→0”) with a certain probability p_D (destruction strength “D”). The probability for this transition is minimal ($p_{D,0}$) when all neighbouring cells are in the state “1” and it increases with every neighbour in the state “0” (see Eq. (2)). Isolated nuclei are most unstable.

Finally, in order to reproduce the observed annular patterns the destruction probability is designed as a dynamic parameter which increases with the number “$N_{n/d}$” of nucleation/destruction events that have occurred in a given cell (x,y) with progressing biasing time (see Eq. (3).). The potential physical background is a change in the conditions of the Ir surface for prolonged biasing treatment.

$$p_N(x,y) = \sum_{|v|\neq|w|} S \cdot N(x+v,y+w) + \sum_{|v|=|w|} S \cdot A \cdot N(x+v,y+w) \quad (1)$$

$$p_D(x,y) = p_{D,0} + \sum_{|v|\neq|w|} D \cdot (1 - N(x+v,y+w)) + \sum_{|v|=|w|} D \cdot A \cdot (1 - N(x+v,y+w)) \quad (2)$$

$$p_D(x,y) = p_{D,0} + 1 - \frac{1}{D \cdot N_{n/d}(x,y) + 1} \quad (3)$$

with $v,w \in \{-1,0,1\}$ or $v,w \in \{-2,-1,0,1,2\}$ when only first or up to second nearest neighbors are taken into account, respectively (with $v,w \neq 0,0$).

A schema of the different steps of the MC simulation is shown in Fig. 2. The results are plotted in Fig. 3. In the first row of Fig. 3, homogeneous patterns with smooth edges are shown. They are obtained for a low and constant value of “D”. In the framework of our simple model, anisotropy could only be obtained when second nearest neighbours were taken into account.

In the second row of Fig.3 ((e) - (h)), patterns are shown for increasing destruction parameter “D”. It is remarkable to see that a rather limited variation of “D” between 0.04 and 0.054 causes a dramatic change from homogeneous to highly branched fractal like patterns. Above 0.054 destruction completely dominates and all nuclei disappear.

In the lower row of Fig. 3 ((i) – (l)) the corral shape patterns could be reproduced. In conclusion, our simple MC simulation which only assumed a dynamic competition between nucleation and destruction events, could reproduce a large variety of features of the domains observed by us and other groups in BEN experiments on Ir.

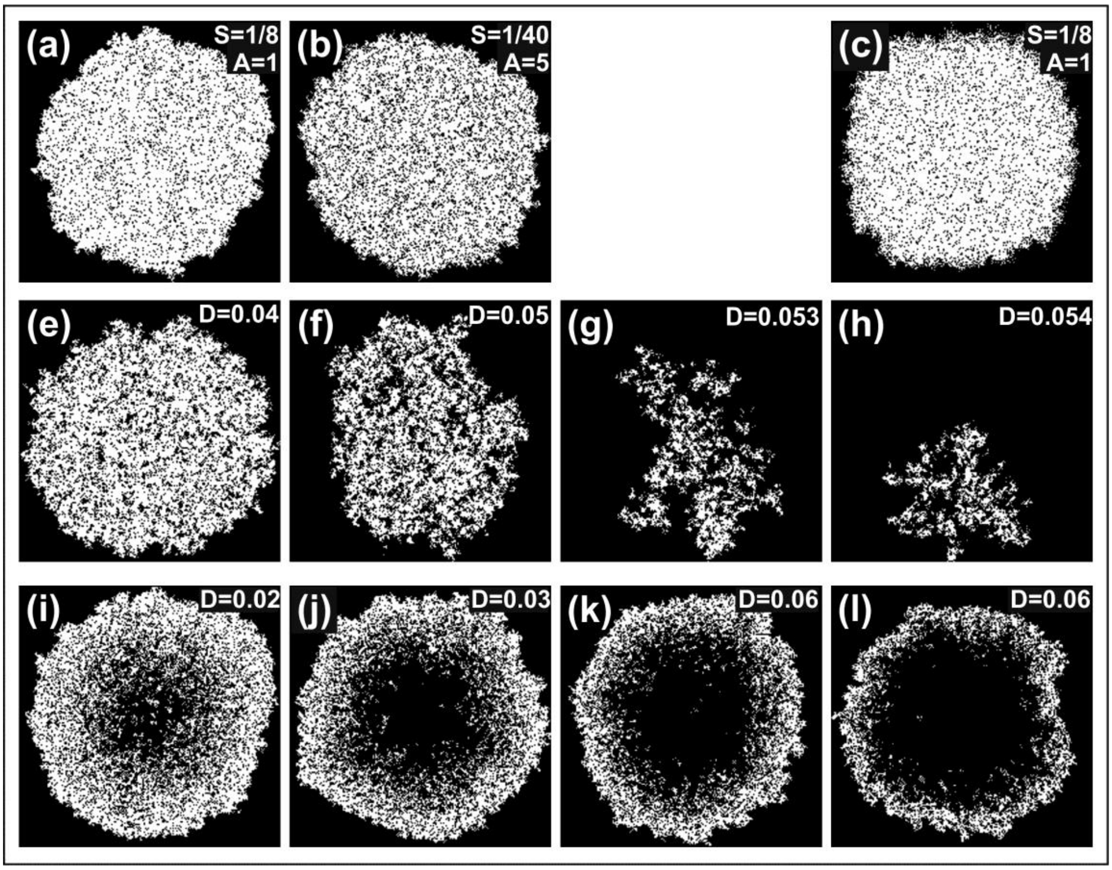

Figure 3: Results of the MC simulation. (a), (b) Shape of final patterns that developed for different nucleation strength S, anisotropy parameter A and a constant destruction parameter D = 0.1. (c) Final pattern obtained by simulation as before but with first and second nearest neighbours contributing to nucleation. (e) - (h) Final patterns obtained from MC simulations in case of isotropic destruction for different values of the parameter D as indicated in the figure. (i) – (l) Final patterns obtained for dynamic destruction depending on the number of preceded nucleation/destruction events. Further parameters are S = 1/8, A = 1.

2. Transition from highly oriented heteroepitaxial grains to a single crystal

The wafer shown in the main article was synthesized by first nucleating heteroepitaxial diamond on Ir/YSZ/Si(001) followed by growth over several days in a high power 915 MHz microwave setup. During growth, the heteroepitaxial diamond layer passes through different stages as schematically shown in Fig. 4: First isolated crystallites grow and merge to form a closed layer. This layer forms a mosaic crystal in which single crystals are separated by a polygonized network of grain boundaries. During growth of the first microns, the grains coarsen but the network persists. Before reaching a thickness of 30 µm, the network dissolves into isolated bands of dislocations. Growth proceeds as a single crystal accompanied by a continuous reduction of the dislocation density.

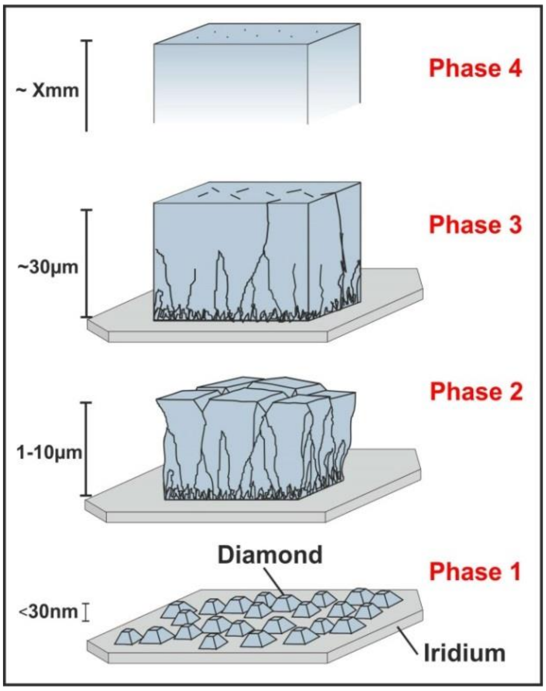

Figure 4: Schema of the different stages of epitaxial diamond growth on Ir: Phase1: nucleation and growth of initially isolated diamond crystals which merge to a closed layer within 30 nm growth. Phase 2: single crystal grains are separated by a polygonized network of grain boundaries typical for the structure of a mosaic crystal. Phase 3: below a thickness of 30 µm a structural transition occurs in which the network dissolves to form isolated bands consisting of dislocations. Phase 4: growth progresses as single crystal and the dislocation density decreases ~ 1/d with d being the layer thickness[9].

3. Structural properties of the single crystal diamond wafer shown in the main article

In order to characterize the structural quality and homogeneity of the wafer shown in Fig. 5 of the main article, rocking curves have been measured along two perpendicular lines across the wafer (see Fig 5 (a) below). The average value and the standard deviation provide quantitative measures for the structural quality and the homogeneity. In Fig. 5 (b) these values are plotted in a diagram taken from Ref. 10. According to the nitrogen content (< 10 ppm) the wafer is classified as type IIa[11]. The comparison with the rocking curves of natural IIa crystals clearly shows that the wafer is structurally in the range typical for single crystals of this type.

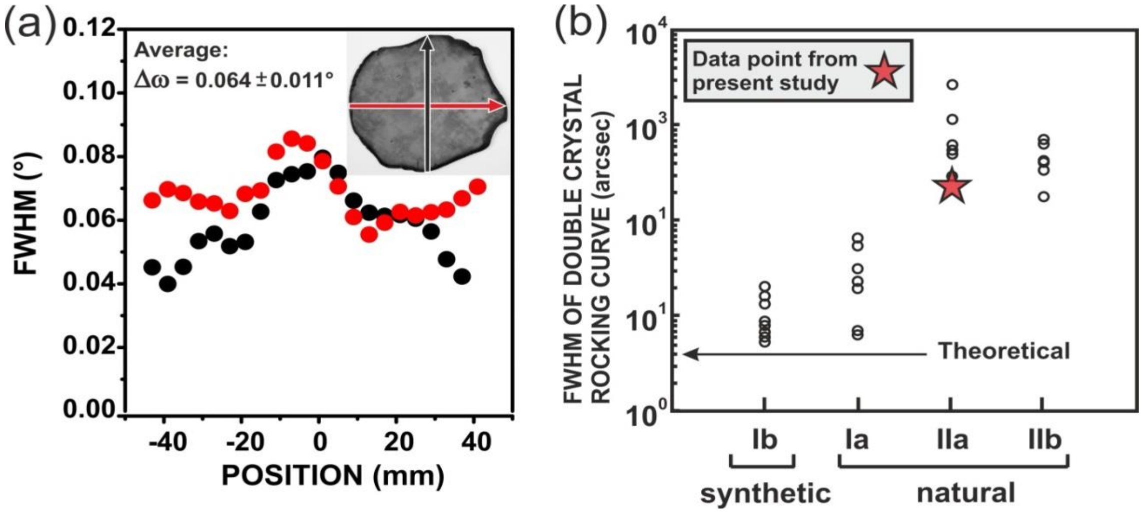

Figure 5: Quality and homogeneity of single crystal diamond disc:

(a) FWHM values of rocking curves measured along two perpendicular lines across the wafer.

(b) Average value of rocking curves (0.064±0.011° = 230±39”) plotted in the graph redrawn from Ref. 10.

REFERENCES

[1] Hwang, R.Q., Schröder, J., Günther, C. & Behm, R. J. Fractal Growth of Two-Dimensional Islands: Au on Ru(0001), *Phys. Rev Lett.* **67**, 3279 – 3282 (1991).

[2] Bott, M., Michely, T. & Comsa, G. The homoepitaxial growth of Pt on Pt(111) studied with STM, *Surf. Sci.* **272**, 161 – 166 (1992).

[3] Geng, D. *et al.* Fractal Etching of Graphene, *J. Am. Chem. Soc.***135**, 6431 - 6434 (2013).

[4] Wang, L. *et al.* Growth and Etching of Monolayer Hexagonal Boron Nitride, *Adv. Mater.* **27**, 4858 - 4864 (2015).

[5] Gsell, S., Schreck, M., Benstetter, G., Lodermeier, E. & Stritzker, B. Combined AFM-SEM study of the diamond nucleation layer on Ir(001), *Diamond Relat. Mater.* **16,** 665 - 670 (2007).

[6] Chavanne, A., Barjon, J., Vilquin, B., Arabski, J. & Arnault, J.C. Surface investigations on different nucleation pathways for diamond heteroepitaxial growth on iridium, *Diamond Relat. Mater*. **22**, 52 – 58 (2012).

[7] Bednarski, C., Dai, Z., Li, A.-P. & Golding, B. Studies of heteroepitaxial growth of diamond, *Diamond Relat. Mater.* **12**, 241 - 245 (2003).

[8] Regmi, M., More, K. & Eres, G. A narrow biasing window for high density diamond nucleation on Ir/YSZ/Si(100) using microwave plasma chemical vapor deposition, *Diamond Relat. Mater.* **23**, 28 - 33 (2012).

[9] Stehl, C. *et al.* Efficiency of dislocation density reduction during heteroepitaxial growth of diamond for detector applications, *Appl. Phys. Lett.* **103**, 151905 (2013).

[10] Fujii, S., Nishibayashi, Y., Shikata, S., Uedono, A. & Tanigawa, S. Study of various types of diamonds by measurements of double crystal x-ray diffraction and positron annihilation, *J. Appl. Phys.* **78**, 1510 - 1513 (1995).

[11] Balmer, R.S. *et al.* Chemical vapour deposition synthetic diamond: materials, technology and applications, *J. Phys.: Condens. Matter* **21**, 364221 (2009).
